# Expanding Possibilities for Intervention against Small Ruminant Lentiviruses through Genetic Marker-Assisted Selective Breeding

**DOI:** 10.3390/v5061466

**Published:** 2013-06-14

**Authors:** Stephen N. White, Donald P. Knowles

**Affiliations:** 1 Animal Disease Research Unit, Agricultural Research Service, US Department of Agriculture, Pullman, WA 99164, USA; E-Mail: Don.Knowles@ars.usda.gov; 2 Department of Veterinary Microbiology and Pathology, Washington State University, Pullman, WA 99164, USA

**Keywords:** small ruminantlentivirus, susceptibility, marker-assisted selection, sheep, goats, *TMEM154*

## Abstract

Small ruminant lentiviruses include members that infect sheep (ovine lentivirus [OvLV]; also known as ovine progressive pneumonia virus/maedi-visna virus) and goats (caprine arthritis encephalitis virus [CAEV]). Breed differences in seroprevalence and proviral concentration of OvLV had suggested a strong genetic component in susceptibility to infection by OvLV in sheep. A genetic marker test for susceptibility to OvLV has been developed recently based on the *TMEM154* gene with validation data from over 2,800 sheep representing nine cohorts. While no single genotype has been shown to have complete resistance to OvLV, consistent association in thousands of sheep from multiple breeds and management conditions highlight a new strategy for intervention by selective breeding. This genetic marker-assisted selection (MAS) has the potential to be a useful addition to existing viral control measures. Further, the discovery of multiple additional genomic regions associated with susceptibility to or control of OvLV suggests that additional genetic marker tests may be developed to extend the reach of MAS in the future. This review will cover the strengths and limitations of existing data from host genetics as an intervention and outline additional questions for future genetic research in sheep, goats, small ruminant lentiviruses, and their host-pathogen interactions.

## 1. Small Ruminant Lentiviruses: Background & Existing Intervention Strategies

### 1.1. Background on Small Ruminant Lentiviruses

Historically, small ruminant lentiviruses (SRLV) were defined by infection and disease in host species, specifically sheep and goats. The history of ovine lentivirus (OvLV), also known as maedi-visna or ovine progressive pneumonia virus, have been covered in detail [1,2,3] and elsewhere in this issue. Similarly, caprine lentivirus (CaLV), also known as caprine arthritis encephalitis virus (CAEV), has been ably reviewed elsewhere [2,3,4]. More recently, it has been shown that these viruses of sheep and goats may be better described more broadly as small ruminant lentiviruses (SRLVs) [2], in part because each virus infects both host species [5,6]. For the rest of this review, we will use OvLV to refer to SRLV infecting sheep hosts and caprine lentivirus (CaLV) to refer to SRLV infecting goat hosts.

Small ruminant lentiviruses are common and inflict economic losses in livestock production. Ovine lentivirus infection is highly prevalent in most sheep-producing countries, including the USA, Canada, most European countries, India, China, Japan, and multiple countries across both South America and Africa [1,2,7,8,9,10,11]. Sheep with OvLV most often develop an interstitial pneumonia, though a range of other manifestations do occur less frequently [2,3,12,13]. Clinical symptoms include varying degrees of dyspnea, mastitis, cachexia, arthritis, and/or encephalitis [2,3,12,14,15]. Production losses for producers stem from early lamb mortality [16], lower lamb weights in older infected ewes [16,17], estimated early culling approximately 1 year prior to uninfected animals [18], and export restrictions [12]. All of these contribute to the high economic cost of OvLV [19,20].

Similarly, CaLV occurs in many countries around the world [2,3,4]. The symptoms of CaLV are predominantly swelling of the carpal joints and interstitial mastitis, although lymphadenopathy, interstitial pneumonia, and encephalitis do occur [2,3,12,21]. Economic losses are due to reduced milk yield, mastitis, interaction with bacterial mammary infection, and premature culling [22,23,24,25]. Importantly, both OvLV and CaLV show evidence of breed differences in susceptibility [26,27,28,29,30,31,32,33,34], that together with other evidence [35,36,37,38,39] suggest a host genetic component in susceptibility and control. 

### 1.2. Existing Intervention Strategies for Small Ruminant Lentiviruses

Many advances have been made in developing intervention strategies against SRLV. One effective strategy is diagnostic testing followed by culling SRLV positive individuals [4,40]. Another effective strategy is separation of lambs/kids at birth to prevent contact and transmission [4,41]. While some vertical transmission of OvLV does occur [42,43], it is the exception rather than the rule and diagnostic testing can often identify such individuals quickly. Both of these strategies have been used to establish SRLV-free flocks and herds [12,44]. 

Another intervention strategy with some effectiveness for OvLV has been extensive rearing [45,46,47]. Presumably, this strategy is effective because of reduced exposure to infectious virus. Regardless, it has been consistently associated with reduced prevalence [45,46,47].

Other intervention strategies that have been or could be tried for SRLV include vaccines and drug treatments. Multiple vaccines have been tried for SRLV, but all have had limited efficacy and some led to increased viremia and disease [3,12,13]. Unfortunately, that has been typical of many vaccines against lentiviral agents [48]. Furthermore, drug treatments exist for some lentiviral agents, such as highly active anti-retroviral treatment (HAART) for use against HIV [49], and could theoretically be used for SRLV. However, drug treatments are generally not available for SRLV at present and would not likely be cost-effective even if they were.

Using the range of available intervention strategies for SRLV, eradication programs have been established in many countries, and these programs have met with some success [12,50]. However, the successes have come with costs, especially for cull replacements, construction and maintenance of separate enclosures for infected or SRLV-free animals, and diagnostic testing. Considering such costs, some countries and producers have opted not to pursue SRLV eradication. Especially for large-scale producer operations, the most effective intervention strategies can have impractical costs [51], leaving only partially effective management adjustments to reduce exposure for such operations. There is a clear opportunity for additional intervention strategies against SRLV, especially those that scale well to large producer operations.

### 1.3. Advantages of Genetic Marker Tests as Interventions for Small Ruminant Lentiviruses

Historically, selective animal breeding has been based on choosing those individuals with desirable phenotypic traits for breeding, in the hope of producing a next generation of animals with a higher frequency of desirable phenotypic traits. That process is dependent on the ability to measure phenotypic traits in the parental generation. For traits like milk yield, body weight, and hair color, those measurements are readily available. However, some other high value traits are more difficult to measure directly on each individual, so a highly correlated proxy for the phenotype may be desirable for these traits. Examples would include carcass traits like steak tenderness potential (that cannot be measured on live animals) and susceptibility to infectious disease (that cannot be measured apart from exposure to infectious agents). In cases like these, the use of genetic markers with consistent trait association to determine which animals are used for breeding may dramatically improve the average rate of trait improvement per generation [52,53]. 

Such a marker-assisted selection (MAS) strategy for infectious disease susceptibility traits has multiple advantages over direct phenotypic measurement. As discussed above, ease of measurement can be a substantial advantage, particularly when healthy animals do not have to be exposed to pathogenic agents. Another potential advantage relates to population health. A small fraction of individuals are responsible for a disproportionate fraction of total population transmission events for a wide variety of pathogens. In general, the 20/80 rule suggests roughly 20% of individuals are responsible for approximately 80% of transmission for many infectious pathogens [54,55]. Experimental data confirm the principle of disproportionate transmission risk in many widely diverse pathogens including respiratory agents, sexually transmitted pathogens, helminthic parasites, and vector-borne diseases [55,56,57,58,59,60,61]. Thus, if MAS could identify and reduce the frequency of the most susceptible, high transmission individuals prior to exposure or transmission, it is possible for MAS to have disproportionate benefit in reducing population-level susceptibility to infectious agents.

Additional advantages of MAS include the avoidance of drug use and scalability with flock size. First, MAS does not involve drug use of any kind. This can be appealing on its own, and it avoids the issue of pathogens developing resistance to drugs that might be used in other species, such as humans. Further, genetic marker use scales well with flock size so that even large flocks can undergo MAS. As with any intervention, there is more labor input with larger numbers of animals, but the effort required is manageable for many operations. In some cases, the cost can be reduced greatly to improve the cost-benefit by sire-only genotyping and selection. The population genetic gain is slower with sire-only selection, but there is a large reduction in numbers of samples and cost required. The genetic gain relative to selection of both sires and dams is related to the mode of inheritance for the desirable allele(s). There is still relatively rapid genetic gain with sire-only selection for dominant markers, but additive markers achieve slower gain and recessive ones achieve greatly reduced gains per generation with sire-only selection. Nonetheless, sire-only selection may have a place in many large producer operations.

Of course, genetic marker tests do not have to be used in isolation from other intervention approaches but can complement existing strategies. Genetic markers for probability of infection have application in a broad range of settings. Test and cull procedures do reduce prevalence if the rate of new positives is lower than the rate of culling [62]. By reducing the rate of new positives genetic MAS for reduced probability of infection would enhance the effectiveness of test and cull strategies. There are smaller benefits for separation strategies, but they can be enhanced if the animals selected have lower probability of becoming lentivirus positive since it may provide increased margin for error in the housing requirement to keep the animals separate from virus. Genetic marker approaches can complement existing interventions, as well as being useful even separately.

## 2. Development of Genetic Marker Tests for Small Ruminant Lentiviruses

### 2.1. General Background on Development of Genetic Marker Tests

#### 2.1.1. Definition of Phenotypes

A critical issue in development and use of genetic markers for MAS is the definition of relevant phenotypes. In the case of small ruminant lentiviruses, the probability of becoming infected, and an animal’s control of virus once infected are the two most important general classes of phenotypic traits for potential application in MAS. The simplest way to record probability of becoming infected under field conditions is to use direct measures of the presence or absence of the virus in individuals averaged over a population [63,64,65,66,67,68,69,70,71,72]. Since lentiviruses induce lifelong infection, the probability of becoming infected can also be measured by indirect diagnostic tests such as those based on serology, which have high concordance with direct viral measures [73]. Controlled dose experiments would have some advantages in simplifying the important exposure variable in evaluating host susceptibility, and such experiments can address possible conditions not encountered frequently in the field. However, natural exposure conditions are of more interest to most producers, and the use of larger numbers of animals in naturally infected flocks can address this question. In fact, that has been one predominant mode of inquiry into probability of infection to date [34,74,75,76,77,78].

An animal’s control of small ruminant lentiviral replication has been measured in two related ways. The first is by direct measurement of proviral concentration [73,79,80,81,82] in peripheral blood, specific tissues, or both [83]. Such measurements have the advantage of reflecting viral replication. Another measure of control is scoring to reflect the severity of pathological tissue lesions in infected animals [83,84,85]. Such measurements can reflect not only viral activity but also immunopathogenesis, in which immune responses from the host that can cause collateral damage in or near infected cells. However, since proviral concentration in peripheral blood is correlated with lesion severity [83] and provides a live animal test readily applicable to large numbers of animals, it is often used to measure control of viral replication [34,74,77,86].

The use of genetic markers for control of viral replication would provide advantages in multiple scenarios. Many producers might find reduced viral replication and reduced average lesion severity among infected sheep to be useful adjuncts to reducing prevalence, particularly if they could be achieved inexpensively. Some producers with markets in their own countries have concluded the costs of OvLV eradication too high to implement. However, the worst pathology can impede commercial production [16,17,18,19,20], and improved control would be helpful in this situation, as well. 

Many other phenotypic measures are related to SRLV infection and disease progression, and these include important production traits like cumulative lifetime offspring weaning weights, milk yield, culling ages, offspring growth, and clinical signs of SRLV disease, among others. It is critical that such traits be included in selection criteria whenever available to enhance overall genetic gain. Indeed, many producers already use many of these traits in phenotypic selection either directly by incorporation into a formal selection index, or more generally by removing individuals with the most severe clinical disease. Such traits are also important measures for development of markers for marker-assisted selection, and joint consideration of these traits will be important as the field progresses. 

#### 2.1.2. Evidence-Based Stages for Development of Genetic Marker Tests

Given defined phenotypes, development of genetic markers begins with evidence those specific phenotypes have a genetic basis in the species of interest. In order to use marker-assisted selection, there not only need to be genes involved in a trait, but the population needs to possess multiple functional genetic variants affecting those genes. That is, there need to be multiple genetic versions, known as alleles, to select one or more over others in making breeding decisions. For both sheep and goats, multiple reports support breed differences in seroprevalence of small ruminant lentiviruses as evidence for a genetic basis for probability of infection [26,27,28,29,30,31,32,33,34]. For sheep, a twin study indicated that host genetics played a larger role than viral genetic variability in probability of infection in at least one case [36]. For severity of infection, breed differences in sheep further support a genetic basis for proviral concentration [34]. Together, these data suggest functional genetic variants for SRLV traits are present within sheep and goat populations. 

Once a genetic basis is established in the species of interest, genetic marker development proceeds through multiple evidence-based stages (Table 1). For traits where a single gene or mutation may account for the entire phenotype, perfect or near-perfect genotype-trait association is often a minimum requirement to develop a genetic marker. However, most traits are considered complex traits because multiple genes influence the phenotypic outcome, and perfect association is not expected under most such situations even with useful functional variants. Broadly speaking, for complex traits the evidence-based stages are: (1) initial discovery of a genetic association, (2A) confirmation of the genetic association by replication in separate validation animal sets, and (2B) evaluation of potential correlated responses to selection. 

**Table 1:** Evidence-Based Stages for Genetic Marker Development.

Stage	Goal	Types of markers potentially involved	Concerns	Marker-Assisted Selection (MAS)
1	Initial discovery of genetic association	Functional variants, markers in linkage disequilibrium (LD; inherited together) with functional variants, spurious associations	(A) Initial association in one animal set directs additional research. (B) Association could be spurious.	Not recommended
2A	Replication of association in validation animal sets	Functional variants, markers in LD with functional variants ^a^	(A) Do markers have predictive value in the sense of consistent trait association? (B) Are there conditions under which predictive value might break down?	Supported by data for traits with replicated association, avoiding conditions where replication of association fails
2B	Assess potential correlated responses to selection	Functional variants, markers in LD with functional variants ^a^	Are there trade-offs where some traits could be improved at the expense of other traits?	Conditions for use refined by assessment of potential trade-offs

^a^ While it is possible for spuriously associated markers to replicate association in separate animal sets, this is expected to be exponentially less likely with each test. Further, even in the unlikely event that such a marker did replicate association, repeated testing should quickly restrict the conditions under which use would be considered as the number of failed association attempts mount.

Many methods to achieve initial discovery of a genetic association have been used, and such methods continue to evolve [87,88]. One method is to sequence genes with known relevance to SRLV or lentiviral biology in a candidate gene approach. This has the advantage of lower cost and known or suspected biological involvement. The downside is that while genetic variants may exist in the gene(s) of interest, there is no prior evidence that any of the variants are functionally important for the trait(s) of interest. Alternative methods like genome-wide association studies (GWAS) require less a priori biological knowledge and can identify even genes with previously unknown involvement. These methods take advantage of linkage disequilibrium between underlying functional mutations and easily sampled markers in the same chromosomal region. Some downsides of these approaches are the multiple experimental stages required to identify chromosomal regions and then test individual genes, and the expense of the genotyping and later gene sequencing. New methods including whole genome sequencing promise to simplify the experimental process by sequencing all possible genetic variants in one pass. Such methods are currently expensive and the bioinformatics required to analyze such data are complex, but developing [87].

Regardless of methodology used, the initial discovery of a genetic association is often the first published report, but the genetic marker(s) in this stage are not ready for broad use. The initial associated marker could be from one of three major types, only two of which are likely to have broad utility. First, the marker could be a functional genetic variant, such as an amino acid substitution at a functional position, a promoter variant with differential transcription factor binding and differential transcription, a variant that creates or destroys a microRNA target site, or other regulatory variants [53,89,90]. A second possibility is the initial associated marker could be in close physical proximity on the chromosome and therefore inherited together with such a functional variant (known or unknown) [53,90,91]. The tendency to be inherited together is called linkage disequilibrium, and in such a case, the marker is in linkage disequilibrium with the functional variant. However, a third possibility is that the initial associated marker might be a spurious association due to chance in the discovery population. The probability of such an event is controlled by the evidence of association (*p*-value) in the discovery animal set, but testing in multiple scenarios across the world suggests some will be spurious by chance even using traditional *p*-value thresholds.

For use as a predictive genetic marker, the next important question is whether the marker will be associated with the trait(s) of interest in the target population, such as a producer’s own flocks. Stage 2A consists of confirmation by replicating the association in separate validation animal sets and is intended to address this question. This stage tends to eliminate spurious associations and markers in only weak linkage disequilibrium with functional genetic variants. Functional variants and markers in strong linkage disequilibrium across populations tend to show replication of association, and therefore pass this stage [53,90,92]. It is only at this point that genetic markers should be considered for predictive use for the original phenotype(s) of interest. Actual selection experiments are the gold standard, and they involve genetic selection in one or more populations with reductions in prevalence and/or severity of small ruminant lentiviruses. However, these experiments are time-consuming and resource-intensive, and in practice those markers with consistent replication of association are often released as commercial genetic markers for traits of interest. 

It is important to note that genetic markers need not show association in all conditions to be useful. One common cause of failed association is low allele frequency in one or more animal sets. This can result in low statistical power to detect a true association, even if present. However, if sufficient power exists and there is still a lack of association, it is important to refine the conditions under which genetic markers are potentially useful. Ideally, the validation sets would include animals from different breed backgrounds, locations, management conditions, and possibly differing viral subtypes. These criteria permit the formation of informed conditions for genetic marker use that take into account conditions of any failed association studies. 

A third evidence-based stage (2B) of genetic marker development involves potential trade-offs in other traits besides the ones identified in the discovery and replication of association stages. For instance, if genetic gain in reduced susceptibility to small ruminant lentiviruses came at the price of dramatic losses in production efficiency, that could restrict the conditions under which the genetic marker might be usefully employed. Further, there are many scenarios under which reductions in susceptibility to one pathogen could create conditions for an increase in susceptibility to another pathogen [93]. For example, productive immune responses often involve either primarily cell-mediated responses or antibody production, and any functional genetic variants affecting balance between types of responses overall might improve immunity to one set of pathogens but do so at the expense of reduced immunity to another [94]. Even within cell-mediated or antibody types of immune responses, improvements in preset genetic cassettes for either T-cell receptors or antibodies could improve readiness for one pathogen but at the expense of readiness for others [95]. While useful genetic markers for infectious disease susceptibility do exist in multiple systems that largely avoid these issues, such concerns are worth noting especially since the underlying research is often conducted after commercial release of genetic markers for particular traits. The overall aim of genetic selection should incorporate all aspects of production, including susceptibility to infectious disease, and consideration of correlated responses to selection plays an important role in informing overall decision-making.

### 2.2.Example of Genetic Marker Tests for Infectious Disease in Sheep: Classical Scrapie

One of the most well-known and widely used genetic marker sets for reduced infectious disease susceptibility in any mammal system is the strong resistance to classical scrapie in sheep conferred by specific genotypes at the prion gene, *PRNP* [96]. A brief review of the development of this genetic marker system provides a useful example to demonstrate principles involved in development of genetic markers for susceptibility to small ruminant lentiviruses. 

It was long observed that scrapie seemed to run in families [96,97], and the familial aggregation of disease provided important evidence for a genetic basis for susceptibility. Eventually, it was understood that scrapie, like all transmissible spongiform encephalopathies, is a protein only disease with a proteinaceous infectious agent [98]. A single gene encodes the protein [99,100,101,102], and deleting that gene conveyed resistance to scrapie in mice [103,104]. The native conformation is called PrP^C^ for cellular prion protein, but a misfolded conformation, termed PrP^Sc^, is both pathogenic and infectious [98]. 

This raised the possibility that scrapie might be simpler from a genetic point of view, in that functional variants would have to influence this one gene’s sequence, expression pattern, or protein availability. Indeed, important amino acid substitution variants were sequenced from families selected for high and low susceptibility [105,106]. Afterwards, many other protein coding substitutions were identified and reported associated with frequency of disease [107,108,109,110,111,112,113,114]. The three amino acid variants most consistently with scrapie prevalence were A136V (alanine or valine at amino acid position 136), R154H (arginine or histidine at position 154), and Q171R (glutamine or arginine at position 171) [96]. Reporting these variants in order gave a relatively small number of linear combinations observed on haplotypes that reflected the protein molecules produced from the *PRNP* gene; these included ARR, ARQ, and VRQ [96]. Perfect association with absence of disease was reported for ARR/ARR homozygotes [115,116,117], and it took decades until even a handful of scrapie cases were reported worldwide with that genotype [118,119]. Further, there was nearly perfect association even in ARR/ARQ heterozygotes [116]. On the other hand, there was a strong association between genotypes including VRQ and scrapie disease prevalence [106,115,116,117], suggesting susceptibility for such genotypes. 

Many of the research steps can be characterized by the stages of genetic marker development as outlined in Table 2. The initial reports of marker association with disease generated lots of interest, and undoubtedly many producers were tempted to use them for breeding decisions at that point. However, the broader research community made breeding recommendations concerning these markers only after evidence was collected from multiple studies demonstrating consistent association with scrapie prevalence [116,120,121]. Further, the research community continued with ongoing definition of situations under which the genetic marker tests might fail, or not work as well as hoped. The good news in the case of the scrapie test in sheep was that the test worked very consistently, and the very few failures served to prove the rule of how well the genetic marker tests worked the vast majority of the time.

**Table 2:** Example genetic markers for infectious disease in sheep: Classical scrapie.

Species	Trait(s)	Gene	Haplotype & Variant	Effect Size	Mode of Inheritance	Breeds	Management	Locale	Potential Correlated Responses to Selection Examined
Sheep	[Common Reference Haplotype]	*PRNP*	ARQ haplotype Contains: A136 R154 Q171	[Common Reference Haplotype]	[Common Reference Haplotype]	Many	Many	Many	[Common Reference Haplotype]
Sheep	Probability of infection	*PRNP*	ARR haplotype Contains: R171	Strong resistance [115,116,117]	Dominant, except to VRQ haplotype	Many	Many	Many	Many
Sheep	Probability of infection	*PRNP*	VRQ haplotype Contains: V136	Strong susceptibility Often 10-fold increased risk [115,116,117]	Largely Dominant	Many	Many	Many	Many
Sheep	Probability of infection	*PRNP*	AHQ haplotype Contains: H154	3–7 fold risk reduction [115,116,117,122]	Partial Dominant, except to ARR or VRQ	Many	Many	Many	Many

There has also been much research done in the area of potential correlated responses to selection for *PRNP* genotypes, and that work continues to the present. The concern was noted very early that selection for scrapie resistance or susceptibility was only one component of a breeding program and would not be as valuable if tradeoffs had to be made between scrapie susceptibility and other production traits [120]. Accordingly, a large body of research addressed potential genotypic association with production traits including reproductive performance, wool characteristics, growth, body type, and dairy ability, among others [123,124,125,126,127,128,129,130,131,132,133]. Work was even done examining susceptibility to small ruminant lentiviruses [86]. Fortunately, most such studies have found no association, indicating a lack of apparent tradeoffs when making breeding decisions on the basis of *PRNP* genotypes. However, a recent study reported a potentially conflicting association with *PRNP* genotypes and lamb survival [134]. This could lead to real economic tradeoffs in breeding decisions [135], but the association should be confirmed in additional animal sets before breeding recommendations are changed. Indeed, a large study designed to confirm or refute the postnatal survival association with *PRNP* genotypes failed to find confirmatory evidence in more than 38,000 lambs of 10 breeds [136], so no changes in breeding strategy were indicated.

### 2.3. Current State of Genetic Marker Tests in Sheep for Ovine Lentivirus

Like the scrapie case outlined above, the same sets of issues need to be addressed to develop genetic marker tests for susceptibility to and control of small ruminant lentiviruses. There have been several important studies demonstrating involvement of many gene products in SRLV infection and disease [137,138,139,140,141,142,143,144,145,146,147,148,149,150,151,152,153,154,155,156,157,158,159,160,161,162], but these do not specifically relate to individual genetic variants present in sheep. In addition to building understanding of infection and disease, such valuable results prepare the way for further work to address whether such genetic variants exist in small ruminants, and if so, begin developing genetic marker tests. Other helpful literature includes individual association studies performed on various sheep genes and gene regions in connection with OvLV traits, such as *DRB1*, *CCR5*, *TLR7*, *TLR8*, *TLR9*, and additional MHC loci [34,74,76,78,84,85], and the many reports of significant association at several genetic loci would pass the first evidence-based stage outlined in Table 1. These, too, are promising research results that advance understanding of host-pathogen interaction, and they advance the field in important ways. These results will be discussed further in Section 3.3. 

However, thus far only variants in the *TMEM154* gene have resulted in consistently replicated association for a validated genetic marker test passing the second evidence-based stage in development (Table 3) [75]. At this point, little is known about the functions of the Transmembrane Protein 154 (TMEM154), but the genetic marker test is well demonstrated. In particular, Heaton *et al.* identified the *TMEM154* gene by GWAS and used sequencing methods to detect a range of potentially functional mutations in this gene, including a charged substitution (E35K) in a predicted extracellular domain, a frameshift mutation in codon 4, and another frameshift mutation in codon 82 [75]. As with the *PRNP* gene in scrapie, the most succinct and biologically accurate way to represent the variants may be as haplotypes containing combinations of variants expressed in individual protein molecules. However, given the range of different variant types identified (not all simple amino acid substitutions), Heaton *et al.* used numbers to represent the observed haplotypes [75,163]. The three haplotypes observed at the highest frequencies were named haplotypes 1–3, and phylogenetic analysis showed haplotype 3 was ancestral in ruminants [75]. Haplotype 1 differed from haplotype 3 by a single amino acid substitution, the charged substitution, E35K. Similarly, haplotype 2 differed from the ancestral haplotype 3 only by a single amino acid substitution, N70I. Sheep were taken from flocks with high OvLV seroprevalence (26%–80%) under natural exposure conditions, and individual sheep with at least one copy of either haplotype 2 or 3 were 2.85 times more likely (95% CI 2.36–3.43) to be infected under field conditions than sheep with two copies of haplotype 1 [75]. No difference was observed between haplotypes 2 and 3, suggesting N70I did not play a detectable functional role related to odds of infection with OvLV [75]. These data led to the suggestion that the presence of at least one ancestral haplotype (such as haplotype 3 or the closely related haplotype 2) may convey high risk of OvLV infection [75]. Importantly, the association was quite strong (*p* < 0.0001) in the original case-control set from Nebraska, in a second case-control set from Nebraska, and in nine additional cohorts containing many breeds sampled from three states [75]. In total, more than 2,900 sheep were sampled representing 11 breeds [75]. This represents strong evidence for consistent association in a wide range of conditions. 

**Table 3:** Current genetic marker test for small ruminant lentivirus traits.

Species	Trait	Gene/Region	Specific variant	Effect Size	Mode of Inheritance	Breeds	Management	Locations	Potential Correlated Responses to Selection Examined
Sheep	Probability of infection	*TMEM154*	Haplotype 1 Contains: K35 Substitution	Genotypic relative risk: 2 copies confer 2.85-fold reduced risk under field exposure [75]	Possibly Recessive	Columbia, Dorset, Finnsheep, MARCIII, Rambouillet, Romanov, Suffolk, Texel, Dorper ^a^, Katahdin ^a^	Extensive, Semi-intensive with variable levels of confinement during lambing	Nebraska, USA Iowa, USA Idaho, USA	Research needed
Sheep	Research needed	*TMEM154*	Haplotype 4 Contains: Codon 4 Frameshift	Research needed	Research needed	Research needed	Research needed	Research needed	Research needed
Sheep	Research needed	*TMEM154*	Haplotype 6 Contains: Codon 82 Frameshift	Research needed	Research needed	Research needed	Research needed	Research needed	Research needed

^a^ These breeds were represented by less than 50 individuals in existing studies [75].

The currently available data on effect size, allele frequencies, and apparent mode of inheritance have many positive implications for the use of this *TMEM154* genetic marker test. First, the effect size for odds of OvLV infection under field conditions is quite large, but does not represent complete resistance. This may seem strange to some who have been accustomed to the strong resistance to classical scrapie provided by the ARR haplotype, but complex traits (multiple genes involved) are likely to be the norm for most traits of commercial interest, including susceptibility to infectious disease. Within the realm of complex trait genetics, the effect size of 2.85-fold relative risk is not only a very useful difference for producers, it is also quite large for a single gene. This implies a 185% excess risk of OvLV for animals with at least one copy of haplotype 2 or 3. A relatively well-characterized complex trait is human type 2 diabetes, for which at least 40 validated loci have been found [164]. Among these, the largest effect sizes are in the range of about 1.40 [164,165,166,167,168]. Even with some differences in effect size calculation (allelic *versus* genotypic), those estimates are still considerably smaller than that of *TMEM154* with OvLV. The *TMEM154* genetic marker test consistently provides a very useful, large difference in odds of OvLV infection. 

The use of genetic marker tests depends on allele frequency because it affects the speed and ease at which genetic gain can be made in a population. The favorable haplotype 1 was observed at relatively high frequency but was still the minor allele in most populations studied [75]. This suggests relatively rapid genetic progress could be made by elevating the frequency of haplotype 1 in these populations because (1) the favorable allele exists at relatively high frequency, implying breeding individuals with haplotype 1 could be identified readily and (2) haplotype 1 was not at very high frequency, which would preclude much potential genetic progress based on this locus. Further, haplotype 1 was present in many breeds [75], suggesting that this haplotype may be widespread among domesticated sheep. This would enable genetic progress in most populations without the need for time-consuming steps like introgression of the allele through multiple generations of marker-assisted backcrossing [169,170], which is additional good news for the use of this genetic marker test as an intervention for OvLV.

Mode of inheritance is another factor with implications for the rate of genetic gain possible using a genetic marker test for MAS. In the case of the current *TMEM154* test, the favorable haplotype 1 appeared recessive to the less desirable haplotypes 2 and 3 [75], though additional work will establish actual mode of inheritance across generations within families. If confirmed, recessive inheritance will slow rates of genetic gain in most situations, because progress will be measured by proportions of animals possessing two copies of haplotype 1, rather than only a single copy. Since individuals will need to inherit from both sire and dam, one option will be to genotype entire flocks and carry out MAS on both rams and ewes. This will enable quite rapid genetic progress in most situations, at the expense of additional labor and cost to genotype the ewes, especially in large flocks. Alternatively, the labor-saving and lower cost option of sire-only selection will also work, albeit more slowly. Depending on the initial allele frequency of haplotype 1 in the flock, it may take up to twice as many generations to achieve the proportion of lower susceptibility individuals possible if the favorable allele had dominant inheritance because consolidation generations may be necessary to get individuals with copies from both parents. In summary, the large effect size and high minor allele frequency for haplotype in a wide range of breeds suggest widespread genetic progress in susceptibility to OvLV is possible with the *TMEM154* genetic marker test, and the recessive mode of inheritance for the favorable haplotype 1 creates a tradeoff for producers between rapid genetic gain at higher cost, or somewhat slower gain at lower cost.

## 3. Future Opportunities and Needs

The exciting developments to date not only provide a meaningful intervention for OvLV, they also raise many other important questions. Some of these questions relate to the further development of genetic marker tests as interventions for small ruminant lentiviruses. Other questions are broader and regard the nature of functional roles of both host and viral components in host-pathogen interactions. Below, we review some important research needs and opportunities in these areas.

### 3.1. Continued Development of Existing Genetic Tests Based on TMEM154

The existing genetic marker test in sheep is based on the three most common haplotypes at *TMEM154*, and more can be done to develop this test. While the test has been validated in many sheep of diverse breeds and management locations, further testing may continue to expand the range of conditions under which use of the test is supported by data. Alternatively, there may be situations in which the test is less helpful. The extent of current data suggest those situations may be very limited, but if they are identified it will be important to address potential reasons. Those could include differing breeds, locations, management conditions, and possible viral adaptations to compensate for host defenses, among others.

A second important issue in the continuing development of the current genetic marker test based on *TMEM154* haplotypes 1–3 regards potential correlated responses to selection using this test. A critical need will be studies to assess potential correlated responses to selection involving production traits. There is little evidence of loci associated with production traits located on sheep chromosome 17 near the *TMEM154* gene in the current SheepQTL database [171,172], so the very preliminary outlook does not include large concerns about specific traits. However, the studies underlying the SheepQTLdb are not expected to represent all important genes or all important production traits, and it will be important to generate data to confirm or refute the existence, direction, and magnitude of any potential correlated responses in production traits. 

Also, as with any genetic marker test for susceptibility to infectious disease, another area of interest for potential correlated responses to selection is other infectious disease traits. A simple example would be association with other SRLV traits, and the literature so far has not addressed whether the current *TMEM154* genetic marker test has a relationship with control of OvLV, in addition to odds of infection. Further, it is possible that genetic improvement in susceptibility to one agent may improve or decrease susceptibility to other agents or other traits related to the same agent, and clear examples have been documented, including with the CCR5 delta-32 mutation and HIV *versus* West Nile Virus [173,174,175,176,177]. An important question will be whether the current *TMEM154* genetic marker test has a relationship with highly prevalent and economically important helminth parasites of small ruminants, but there are many other infectious agents that will also be of interest. These are early days in assessing the potential relationship of current intervention tools based on host genetics to other traits in sheep.

Additional opportunities with *TMEM154* in sheep concern the additional haplotypes identified by Heaton *et al.* that were not observed at sufficient allele frequencies to perform meaningful tests of potential resistance (Table 3) [75]. It is possible that one or more of these alternate haplotypes could confer a greater advantage, possibly including strong resistance. Haplotype 1 used in the current test differs from the ancestral haplotype by a charged amino acid substitution of lysine for glutamic acid at position 35 in the TMEM154 peptide. While this is likely to change the conformation of the TMEM154 protein, some of the low frequency haplotypes are predicted to alter TMEM154 protein structure much more drastically. For example, haplotype 4 has a frameshift mutation at amino acid position 4, which is within the signal peptide used to direct the final protein to its cellular destination. This would be essentially a natural knockout mutation of the *TMEM154* gene, and it was observed as one of the more common minor haplotypes [75]. There was also another frameshift mutation (at position 82) on the very rare haplotype 6 that would encode only a partial TMEM154 peptide. Either of these may have more extreme properties than haplotype 1. There was also a haplotype 10 that was very similar to haplotype 1, but with an additional charged amino acid substitution of histidine for leucine at position 14. Additional work will be required to assess the relative susceptibility to OvLV and potential correlated responses to selection of animals bearing these haplotypes. 

A further opportunity exists in assessing potential relative susceptibility to CAEV of goats with different *TMEM154* haplotypes. It is known that some *PRNP* haplotypes are shared between sheep and goats, though certainly not the majority of them [111,178,179,180,181]. It is currently not known whether the sheep *TMEM154* haplotype 1 exists in goats, or whether it might have similar association with CAEV. It is also not known whether other haplotypes, shared or unique to goats, may play an important role in CAEV susceptibility, but these are important questions underlying the development of genetic marker test interventions for this economically important pathogen. As always, potential correlated responses to selection will be an additional dimension of needed work in goats.

### 3.2. Expanded Virus/Host Interaction Data

The discovery of *TMEM154* genetic association validated in many populations has possible implications extending beyond genetic marker tests into biology of virus-host interactions. In particular, it is possible that TMEM154 protein serves as a viral receptor or co-receptor to enable OvLV entry into host cells [75]. In humans, CCR5 is a co-receptor for HIV and the delta-32 deletion mutation eliminates the necessary co-receptor from the cell surface [176]. In so doing, this mutation provides strong resistance against HIV infection [175,176,177]. Even highly exposed individuals with two copies of the delta-32 mutation remained HIV negative after repeated exposure to the HIV virus [176]. It is interesting to note that this mechanism efficiently provides strong resistance to HIV at low to no energy cost for the host. If TMEM154 protein were a receptor or co-receptor for OvLV, it might not only enable energy-efficient resistance, but may also provide many other insights on viral function and host-virus interaction. 

Existing data suggest that TMEM154 protein will not be the only receptor for SRLV, but it may play an important role as a receptor for some strains and/or as a co-receptor for many or all SRLV. Specifically, somatic cell hybrid experiments identified a receptor for strain 1514 on ovine chromosome 3p in a region not known to harbor receptors for any other lentivirus [182]. A separate study on strain EV1 identified the receptor location on mouse chromosome 2 or 4 [183]. While *TMEM154* is conserved among mammals, neither of these locations are consistent with the location of *TMEM154* on ovine chromosome 17 [75] and mouse chromosome 3 [184]. However, it has also been shown by both antibody-based competitive virus exclusion and cell fusion experiments that not all SRLV share the same receptor [185]. If TMEM154 protein is a receptor or co-receptor for OvLV, then it would add another protein and potential drug target to the list of known lentiviral receptors. If not, it will be interesting to learn what other roles TMEM154 protein may have in SRLV infection that account for the consistent genetic association [75]. 

On the other side of the virus-host interaction, additional questions relate to viral genetic factors that interact with host *TMEM154* genotype in probability and/or control of viral replication. Many SRLV genetic variants have been identified [14,186,187,188,189,190,191,192,193,194,195,196,197,198,199], and some are known to influence various aspects of viral function and host pathology. These include a duplication [192] and a separate deletion [190,191] in the long terminal repeat (LTR) region that acts as a promoter region for the virus [200,201,202,203,204]. The duplication upregulates transcription and is associated with neurovirulence [192]. The deletion is associated with reduced lung pathology [191]. These or other viral variants may influence probability of infection in animals of differing *TMEM154* genotypes, and in the long term it is possible that viral variants might emerge to mitigate the partial resistance currently offered by these genotypes. However, additional research is needed to investigate these possibilities.

### 3.3. Additional Genetic Tests

Additional opportunities exist to produce genetic marker tests based on other genes besides *TMEM154*. Most traits have a complex genetic architecture with many genes involved, and unlike scrapie, more than one genetic marker test will need to be used to address most traits of value to producers. The use of multiple genetic marker tests presents little problem to the producer because one DNA sample can be used for multiple tests, so a single sample submission can still lead to a combined estimate of susceptibility. This is a common occurrence for many genetic marker tests, from heart disease susceptibility in humans to production traits in livestock. 

Several lines of evidence suggest additional genetic marker tests based on host genes other than *TMEM154* will be possible. Many initial genetic associations have been reported with differential odds of infection and/or control (Table 4) [34,74,77,84,85]. These include high quality work on MHC gene and MHC region associations, including susceptibility alleles potentially similar to those used successfully in marker-assisted selection for other infectious disease traits [205]. There have even been multiple associations in the same gene, such as *MHC-DRB1* [34,84]. However, the underlying variants have not yet been validated replicated association; to date, allele frequency differences between flocks tested have precluded such replication [34,84]. There has also been systematic genome-wide association for both odds of infection and proviral concentration, where many additional genomic regions were detected at similar or better association than the *TMEM154* region [77]. Further, TMEM154 protein is not the only cellular receptor for SRLV [182,183], and it is known that SRLV co-infect and recombine widely [2,206]. The initial development of additional genetic marker tests would require a validation step to replicate the associations in validation animal sets, but the preliminary evidence is promising that this will be possible for multiple additional genetic regions.

**Table 4:** Genetic variants with reported initial associations for small ruminant lentivirus traits.

Species	Trait	Gene/Region	Specific variant	Breeds	Locations	Reference
Sheep	Probability of disease	*MHC-Class I*	OMHC1*205	Latxa	Araba, Spain Gipuzkoa, Spain	[85]
Sheep	Probability of disease	*MHC-DRB2*	DRB2*275	Latxa	Araba, Spain Gipuzkoa, Spain	[85]
Sheep	Probability of disease	*MHC-DRB1*	Haplotype *0325	Latxa	Araba, Spain Gipuzkoa, Spain	[84]
Sheep	Proviral concentration	*MHC-DRB1*	Haplotypes *0403 and *07012	Rambouillet, Polypay, Columbia	Idaho, USA	[34]
Sheep	Proviral concentration	*CCR5*	rs119102753	Rambouillet, Polypay, Columbia	Idaho, USA	[74]
Sheep	Probability of infection	*DPPA2, DPPA4*	OAR1_185953850	Rambouillet, Polypay, Columbia	Idaho, USA	[77]
Sheep	Probability of infection	*SYTL3, GTF2H5, DYNLT1, TMEM181, EZR*	OAR8_88021348	Rambouillet	Idaho, USA	[77]
Sheep	Proviral concentration	*C19orf42, TMEM38A, NWD1, MED26, SLC35E1, CHERP*	s27054	Polypay	Idaho, USA	[77]
Sheep	Proviral concentration	*ZNF192, ZSCAN16, ZNF165, ZNF389*	s65956	Rambouillet	Idaho, USA	[77]

In fact, there may be advantages to the simultaneous use of multiple underlying genetic marker tests, especially for infectious disease traits. If each sheep or goat genetic marker test provides a hurdle to infection, replication, or transmission, then the use of only one test implies the virus must overcome only one hurdle to achieve heightened infectivity or replication. This can be done by one or more mutations that enable the virus to evade a given host defense, and the overall phenomena is often described in terms of an arms race [207]. Since viruses (and most infectious agents) mutate more quickly than the host, this gives the virus an advantage in the long-term arms race. However, if multiple hurdles could be erected, then multiple hurdles would have to be cleared for the virus to achieve heightened infectivity or replication. This is the principle behind the combination of multiple forms of partial genetic resistance to wheat rust, among other practical real-world examples [208].

Once multiple genetic marker tests become available, genetic interactions will become an additional research need. Specifically, some forms of partial resistance may produce a genetic interaction in which some combinations produce even stronger resistance (or potentially, weaker resistance) than one would predict from the sum of the individual components [209]. Once multiple tests become available, quantifying such interactions, if any, will become an important research objective. However, the general principle of combining multiple tests (with or without formal genetic interactions) may provide a context in which breeders can counter the advantage possessed by most infectious agents due to their higher mutation rate.

We note that particular caution should be used in interpreting and applying MHC gene associations with infectious disease traits. Such associations are very useful for study of host-pathogen interaction and antigen presentation. However, there are additional concerns related to correlated responses to selection when using MHC-based genetic marker tests for marker-assisted selection. First, since the classical MHC genes are used to present antigens from a diverse variety of sources, it is possible that selection toward homozygosity for one allele might diminish the immune system’s ability to respond to other pathogens. Further, the MHC region is characterized by long linkage disequilibrium that spans many immune system-related genes [210,211]. Thus, even if selection of one original variant did not adversely impact susceptibility to other pathogens, a wide variety of variants at other important genes could do so. Nonetheless, successful marker-assisted selection based on MHC loci has been reported by selecting against strong susceptibility loci [205]. Since that strategy reduced susceptibility to a pathogen without dramatically reducing the overall genetic variability across the MHC in the population, it may have wide applicability in many situations. However, we urge caution in using other selection strategies based on MHC genes until potential correlated responses to selection have been examined and shown to be helpful and safe to a very high standard. 

### 3.4. Additional Phenotypes to Address Superspreader Hypothesis with OvLV

One of the potential benefits of MAS applied to infectious disease traits is the possibility of eliminating the highest transmission individuals prior to infection. However, it is still not known how to identify predominant forms of infectious virions for OvLV. There are open questions about transmission through cell-associated virus *versus* free viral particles [212,213,214]. While recent advances have provided important clues [215], there is still no way to reliably measure shedding of OvLV in ways meaningful for natural transmission. The development of such a measure is an important research need to facilitate identification of superspreader individuals. Further, it would greatly assist the development of additional genetic marker tests for high shedding if such a diagnostic measure was amenable to high throughput use for examination of large numbers of animals.

Some argue that selection to dramatically improve the control of viral replication is selection for tolerance to the virus and could increase the difficulty of detecting the lowest proviral concentration positives as infected. In theory, if such low concentration individuals were important sources of transmission, this could slow eradication efforts. However, the relationship between proviral concentration and transmission is unknown. It is possible that higher proviral concentration could indicate not only more provirus within an individual, but also more infectious virus shed from an individual. If so, the superspreaders of OvLV might be individuals with high proviral concentration. As such, there could be disproportionate benefit to selective elimination of these individuals. However, development of tools to measure transmission will be necessary to answer this question.

### 3.5. Additional Studies on Other Small Ruminants

The discussion of future opportunities has thus far focused mainly on sheep, but there are many opportunities to address SRLV in goats using genetic methods. Many studies have noted breed differences among goats in susceptibility to CAEV [30,31,32,33], strongly suggesting a genetic basis. However, few studies have examined specific genetic regions to identify factors underlying these genetic differences, except those focusing on the MHC region using serologic methods rather than modern sequencing to define alternate genotypes [37,38]. We have already discussed possibilities for assessing *TMEM154* in goats, but both other candidate genes and genome-wide approaches may provide insights into additional gene regions and variants associated with CAEV traits in goats. Any genes identified in connection with OvLV may also be of interest in goats with respect to CAEV, especially given the close relationships among SRLV, ability to infect multiple species, and recombination between SRLV. Further, the recent development of large-scale genome-wide genotyping reagents for domestic goats [216] suggests that genome-wide association may provide a tool for identifying further host genes of interest in goats, some of which may be useful in sheep, as well. 

### 3.6. Possibilities for Genomic Selection

Genomic selection refers to a separate, but related, technology compared to the marker-assisted selection described thus far. While marker-assisted selection generally involves smaller numbers (usually less than 100) of markers placed very close to or consisting of functional mutations, genomic selection uses very large numbers (many thousands or more) of markers throughout the genome to assist in making selection decisions [217]. This is made possible by the high density single nucleotide polymorphism (SNP) arrays now available in many species, including sheep and goats [216,218]. Genomic selection requires the use of very large reference populations (generally 1,000s or 10,000s of animals) with relevant phenotypic data used to generate statistical prediction equations [217]. These equations are then used in combination with genotypes only from different animals to generate predictions about genetic merit. The large number of markers can potentially provide greater total predictive ability than the smaller number of markers used in marker-assisted selection, even if the predictive contributions of each marker are much smaller. 

Bovine dairy applications of genomic selection have been among the most successful in livestock species to date [217,219]. Such methods have achieved an estimated doubling in the rate of genetic gain in the special case of dairy cattle breeds with small effective population sizes and long linkage disequilibrium [217]. However, there are more beef breeds, they have shorter linkage disequilibrium, and genomic selection has offered only moderate to small gains in such situations [217]. Genomic selection has been implemented in sheep for some traits [220,221,222], and success has likewise been moderate at best [217]. This may be related to the short linkage disequilibrium in sheep [77,218]. 

In addition to the difficulties in short linkage disequilibrium contexts, the development of genomic selection for infectious disease traits in small ruminants faces several technical hurdles in the near term. The necessity for large reference populations with consistent phenotypic data has been a major limitation for infectious disease traits [219]. While reference populations for genomic selection in sheep are under development, so far none have developed phenotypes for SRLV. One study has included milk somatic cell score (SCC) for dairy sheep, but the genetic correlations of SCC and SRLV traits have not been well-defined in many production situations and such records only exist for dairy breeds. Further, current reference populations contain only a few breeds, and considerable difficulties have been noted in applying prediction across breeds [223,224]. One of the largest costs in developing additional reference populations is genotypes, which currently often cost nearly as much as the value of each animal in small ruminants though costs are projected to decline in time. Finally, additional cost concerns include the need to update phenotypic measurements regularly to prevent degradation of the predictive value across generations.

However, in the long-term genomic selection is a very promising tool for advancing livestock breeding and multiple opportunities exist to integrate infectious disease and health traits into genomic selection for small ruminants. First, the addition of markers validated for marker-assisted selection into genomic selection breeding schemes is feasible. Also, a major current limitation is the density of genotyping arrays. However, a high density array for sheep will be released in the near future that may mitigate this concern. Further, and genotyping by sequencing may soon be economically feasible. Once it is, density of markers will no longer be a constraint and short linkage disequilibrium will be an advantage rather than a disadvantage [217]. In this context, small ruminants may be well-positioned to benefit.

## 4. Conclusions

Intervention strategies for SRLV based on host genetics and genomics have great promise that is only beginning to be fulfilled. There is evidence to suggest more genetic marker tests will follow, and combining tests use may have long-term benefits against rapidly evolving SRLV. However, it is important to understand evidence-based stages of genetic marker test development so producers and other users of these tests are aware of both the strengths and weaknesses of existing evidence and potential types of research developments that may be expected going forward. Finally, increased understanding from host genetics may play an important role in understanding virus-host interactions for SRLV, and potentially for other lentiviruses.
